# Arteriovenous cerebral high-flow shunts: genetic analysis of patients from a pediatric tertiary care center

**DOI:** 10.3389/fgene.2025.1430657

**Published:** 2025-03-28

**Authors:** Ferruccio Romano, Patrizia De Marco, Giulia Amico, Marisa Mallamaci, Marco Pavanello, Gianluca Piatelli, Marcello Scala, Federico Zara, Francesca Faravelli, Mariasavina Severino, Domenico Tortora, Francesco Pasetti, Lucio Castellan, Silvia Buratti, Valeria Capra

**Affiliations:** ^1^ Clinical Genomics and Genetics Unit, IRCCS Istituto Giannina Gaslini, Genoa, Italy; ^2^ Medical Genetics Unit, IRCCS Istituto Giannina Gaslini, Genoa, Italy; ^3^ Human Genetics Unit, IRCCS Istituto Giannina Gaslini, Genoa, Italy; ^4^ Neonatal and Pediatric Intensive Care Unit, Acceptance and Emergency Department, IRCCS Istituto Giannina Gaslini, Genoa, Italy; ^5^ Neurosurgery Unit, IRCCS Istituto Giannina Gaslini, Genoa, Italy; ^6^ Department of Neuroscience (DINOGMI), University of Genoa, Genoa, Italy; ^7^ Neuroradiology Unit, IRCCS Istituto Giannina Gaslini, Genoa, Italy; ^8^ Pediatric Radiology Unit, IRCCS Istituto Giannina Gaslini, Genoa, Italy; ^9^ Neuroradiology Unit, IIRCCS Ospedale Policlinico San Martino, Genoa, Italy

**Keywords:** vein of Galen aneurysmal malformation, arteriovenous shunt, differential diagnosis, next-generation sequencing, genotype–phenotype correlation, vascular remodeling

## Abstract

**Introduction:**

Arteriovenous cerebral high-flow shunts include the vein of Galen aneurysmal malformation (VGAM) and vein of Galen dilatation, which are considered secondary to arteriovenous malformations or arteriovenous fistulas. These entities are often sporadic but are found in association with variants of the *RASA1* and *EPHB4* genes (capillary malformation–arteriovenous malformation, CMAVM; OMIM #608354) or *ACVRL1, ENG,* and *SMAD4* genes (hereditary hemorrhagic telangiectasia, HHT; OMIM #187300). The clinical phenotypes associated with these conditions are highly variable, with incomplete penetrance and mostly dependent on the hemodynamic consequences (including heart failure and cerebral hemorrhage) or management complications rather than anatomical vascular variations *per se*. The present study aimed to genetically characterize a cohort of 29 patients affected by arteriovenous cerebral high-flow shunts who were treated at a pediatric referral center.

**Methods:**

The genetic techniques employed include next-generation sequencing, multiplex ligation-dependent probe amplification, and whole-exome sequencing.

**Results:**

Of the 29 patients, 11 cases were found to have variants in genes associated with vascular functions, five cases received a genetic diagnosis, one case presented with a variant of uncertain significance in the *EPHB4* gene, and five cases showed variants in novel genes possibly linked with cerebrovascular disorders.

**Discussion:**

We provide extensive case descriptions and attempt to infer the genotype–phenotype correlations; variants in all of the known genes associated with arteriovenous cerebral shunts were reported in VGAM patients, while cutaneous angiomas were specific to *RASA1* mutations. The genotypic and phenotypic descriptions of the affected individuals may thus have relevant implications in terms of better pathophysiological understanding, genotype–phenotype correlations, treatment strategies, and outcomes.

## Introduction

Vein of Galen aneurysmal malformations (VGAMs) constitute high-flow arteriovenous shunts that are supplied by the choroidal arterial feeding vessels and drain into the aberrantly persistent median prosencephalic vein of Markowski, which is the embryonic precursor of the vein of Galen (Raybaud et al., 1989; Lasjaunias et al., 2007; Li et al., 2021); it is the most frequent type of intracranial arteriovenous malformation (AVM) in children (Gillet de Thorey et al., 2022). Prenatal diagnosis of this condition is usually made during the third trimester with color Doppler ultrasound. Fetal magnetic resonance imaging (MRI) is generally essential for the study of VGAM features to detect possible associated brain abnormalities and evaluate possible differential diagnoses, which are challenging in the case of arteriovenous cerebral high-flow shunts. Unlike VGAMs, in case of vein of Galen dilatation (VGD), drainage of the AVMs or pial arteriovenous fistulas (AVFs) involve a dilated but already formed vein of Galen (Tas et al., 2022). Pial AVFs result in direct communication between one or more pial arteries and a cerebral vein; they lack an intervening nidus and are located in the subpial meningeal space. The pathophysiological mechanisms of cerebral AVFs remain unclear owing to their rarity (Lasjaunias et al., 2007; Vo et al., 2023). As the angio-architectures and clinical progression of AVFs may differ, careful imaging analysis is necessary for better clinical management (Vo et al., 2023). Newborns with either VGAM or VGD may suffer from rapidly deteriorating high-output heart failure and are at risk for severe neurological complications derived from the high blood flow and pressure; these outcomes include hydrocephalus, intracerebral hemorrhage, venous hypertension, macrocrania, seizures, and psychomotor delay (Vivanti et al., 2018; Buratti et al., 2023). Neurological signs are often not directly related to the AVMs but rather indicate their hemodynamic consequences, including heart failure and cerebral hemorrhage, or treatment complications.

VGAM and VGD are largely sporadic and affect both sexes. Numerous cases of cerebral arteriovenous shunts are known to be hereditary or have a genetic basis. Genetic variants in the *EPHB4* gene are estimated to be present in approximately 10% of arteriovenous cerebral shunts, and the percentage of genetic-related cases of VGAM/VGD in the context of capillary malformation–arteriovenous malformation (CMAVM) or hereditary hemorrhagic telangiectasia (HHT) is considered to be even higher (Tas et al., 2022; Bayrak-Toydemir et al., 2011). The genes associated with VGAM/VGD include *RASA1*, *EPHB4*, *ACVRL1*, *ENG*, and *SMAD4* (Tas et al., 2022; Amyere et al., 2017; Duran et al., 2018; Singh et al., 2022). The clinical presentations are heterogeneous for mutations in each of these genes, and the penetrance is incomplete. *EPHB4* and *RASA1* cooperate in the regulation of vascular development by modulating Ras signaling in the endothelial cells, which is a fundamental driver of VGAM pathogenesis (Vivanti et al., 2018; Zhao et al., 2023). Heterozygous germline mutations of *EPHB4* result in a wide phenotypic spectrum, which also includes non-immune hydrops fetalis with atrial septal defects, cutaneous capillary malformations (CMs), and CMAVM syndrome (Vivanti et al., 2018; Amyere et al., 2017; Martin-Almedina et al., 2016). The *EPHB4* gene encodes receptors with pivotal roles in cell adhesion and migration as well as coordination of angioblast segregation into distinct networks of arteries and veins, thus showing key angiogenic and vascular remodeling actions (Vivanti et al., 2018; Adams et al., 1999). The ligand EphrinB2 and its receptor EphB4 are respectively expressed in arterial and venous progenitors, establishing bonds to guide directional progenitor migration (Vivanti et al., 2018).

The protein RASA1 is a negative regulator of the Ras/MAPK signaling pathway and acts as a regulator of cellular growth, differentiation, and proliferation (Bayrak-Toydemir et al., 2011). Germline monoallelic *RASA1* variants are reported to be associated with CMs, CMAVM, VGAM, and cerebral AVFs (Tas et al., 2022; Saliou et al., 2017). ACVRL1 promotes crosstalk between the TGF-β and Ras signaling pathways by associating with RASA1 via the Dok-1 adapter protein (Zhao et al., 2023). The genotype–phenotype correlations in these cases are elusive; Tas et al. (2022) suggested the involvement of *EPHB4* in true VGAM and *RASA1* in pial/choroidal fistulas, in addition to VGAM. On the other hand, *ACVRL1* was considered specifically for pial AVFs (Tas et al., 2022). Although knowledge regarding the genetic contributions to VGAM is increasing, the underlying genetic causes of most cases remain unknown (Zhao et al., 2023). Moreover, the reasons for the large variabilities in clinical expression and low penetrance are only partially elucidated.

The present study aims to provide a portrait of the genetic findings on a cohort of 29 VGAM/VGD patients at a referral center in Italy. Our analysis is intended to highlight possible variants in genes that are already known to be associated with VGAM or VGD and to detect possible variants of clinical interest in genes that are not reported in affected individuals but may have a function in the vascular physiology. This analysis is likely to have a positive impact on knowledge expansion from the point of view of not only etiology and pathogenesis but also clinical management, risk stratification, treatment strategies, and putative genotype–phenotype correlations. We also present some preliminary data on novel genes that may be possibly connected with vascular diseases that require further evidence.

## Methods

### Study design and settings

This work is a single-center retrospective cohort study. The patients included in the study were recruited at Gaslini Children’s Hospital, Genoa, Italy, which is a major pediatric tertiary care center. The VGAM team at this center is a multidisciplinary group of specialists in Clinical Genetics, Perinatal Medicine, Fetal and Pediatric Cardiology, Neurology, Neonatal and Pediatric Intensive Care, Interventional Radiology, Neuroradiology, and Neurosurgery.

### Patient selection and description

In this retrospective study, we collected genetic data from patients with arteriovenous cerebral high-flow shunts who had undergone genetic analyses at Gaslini Children’s Hospital from January 2011 to February 2024. A total of 29 patients with arteriovenous cerebral high-flow shunts who had received genetic analyses were selected for this study; of these, 25 patients (86%) were affected by true VGAM, while two children (7%) had VGD secondary to AVM, and the remaining two patients (7%) had VGD secondary to AVF. The patients have undergone complete multidisciplinary (clinical, neurological, and anesthesiologic) evaluations, and the clinical data were retrieved from the patients’ electronic charts. The brain magnetic resonance imaging (MRI) with magnetic resonance angiography (MRA) data were reviewed by an experienced pediatric neuroradiologist. The VGAM was radiologically defined as a grossly dilated vein of Galen with multiple arterial feeders from the anterior and/or posterior circulation associated with dilatation of the straight and transverse/sigmoid sinuses. Conversely, the presence of a cerebral AVM or pial AVF draining into a dilated vein of Galen was considered as VGD.

We also report data on the long-term clinical outcomes in surviving patients using the pediatric overall performance category (POPC) scale that was administered at the last follow-up. The POPC scale is used to assess the global functions in daily life through the following scores: 1, normal life; 2, mild disability; 3 and 4, moderate and severe disabilities; 5, coma/vegetative state; and 6, brain death (Fiser, 1994).

### Next-generation sequencing (NGS) and whole-exome sequencing (WES)

Genomic DNA was extracted from the peripheral venous blood sample using the Qiasymphony DNA Midi (Qiagen, Italy) commercial kit following the manufacturer’s protocols. The DNA concentration and quality were both evaluated using the NanoDrop 2000 spectrophotometer (Thermo Scientific, Wilmington, DE, United States) and Qubit^®^ 2.0 fluorometer (Invitrogen, Carlsbad, CA, United States) using the Qubit™ dsDNA HS Assay Kit (Life Technologies).

A custom NGS panel was then created using the IonAmpliSeq Designer tool from Thermo Fisher Scientific (Carlsbad, CA, United States) for sequencing of the entire coding region and 10 bp of the adjacent intronic regions of *EPHB4* (NM_004444.5), *RASA1* (NM_002890.2), *ENG* (NM_001114753.2), *ACVRL1* (NM_000020), and *SMAD4* (NM_005359.6). The target regions were entered into the online tool, and the resulting 123 amplicons with sizes of 125–375 bp were divided into two primer pools. Libraries were then prepared starting from 10 ng of genomic DNA using the AmpliSeq Library Kit 2.0 (Life Technologies) according to the manufacturer’s instructions and barcoded using the IonXpressBarcode Adapter. The final concentrations of the libraries was evaluated using the Qubit^®^ 2.0 fluorometer and Agilent High-Sensitivity DNA Kit (Life Technologies). The libraries were then diluted to 15 ng/mL and pooled together. Template preparation and chip loading were performed on the Ion Chef System (Thermo Fisher), and sequencing was performed on Gene Studio S5 (Thermo Fisher Scientific) using 510 Ion Chips. Base calling was generated using Torrent Suite 3.0 software (Thermo Fisher Scientific) along with tmap-f3 on the Ion Torrent server for further analysis. The bam files were analyzed using Ion Reporter software v.5.16 through a customized pipeline. Human genome build 19 was used as the reference during the alignment.

The variants were considered higher priority if (i) they were predicted to affect protein-coding sequences (including non-synonymous, frameshift deletion, and stop-gain variants in exonic or splicing regions); (ii) they had low frequency in reference databases, with minor allele frequency less than 0.01 based on data in the genome aggregation database (gnomAD v2.1.1). The variants were classified according to the American College of Medical Genetics and Genomics (ACMG) criteria (Richards et al., 2015; Rehder et al., 2021) using the Varsome (Kopanos et al., 2019), Franklin, and ClinVar (Landrum et al., 2016) databases. The variants were reported as pathogenic (P), likely pathogenic (LP), of uncertain significance (VUS), benign (B), and likely benign (LB). The VUSs were interpreted by a multidisciplinary team including clinicians, neuroradiologists, and geneticists to ascertain possible genotype–phenotype correlations (ACGS version 4.01 2020). Therefore, the selected variants were validated via Sanger sequencing and evaluated according to the clinical manifestations of the probands and parental segregation, whenever possible, using high-fidelity platinum master mix (Invitrogen) for polymerase chain reaction (PCR) amplification and BigDye Terminator v1.1 kit (Life Technologies) for sequencing.

WES analysis was performed on nine of the cases, both in singleton probands (four cases) and trios (probands and parents, five cases) using the xGen^®^ Exome Research Panel v1.0 -IDT kit comprising 429,826 probes (approximately 19,396 genes) at the Italian Institute of Technology (IIT) in Genoa, Italy. The FASTQ files were aligned to the GRCh38 human reference genome using the Burrows–Wheeler aligner package version 6.1. Variant calling was performed using the Genome Analysis Tool Kit (GATK4), and the variants were annotated with Annovar (database updated 27 May 2019). Variants with frequencies less than 0.1% in ExAC/GnomAD v2.1.1/1000g2015 were predicted as deleterious or damaging by PolyPhen and SIFT having CADD prediction values of at least 25 were considered. Variants classified as pathogenic, likely pathogenic, or VUS (as defined by the Franklin classification tool based on the guidelines of the American College of Medical Genetics) (Richards et al., 2015) were also analyzed. Furthermore, manual evaluations of the variants were performed using the data reported in public databases, such as ClinVar, OMIM, Mouse Genome Database (MGD), and PubMed.

In addition to the variants in genes that are already reported to be associated with VGAM/VGD, we selected VUSs with deleterious predictions according to bioinformatics tools as well as likely pathogenic or pathogenic variants in genes with roles in vascular homeostasis or functions. The variants were selected for biologic plausibility, demonstrated vascular functions in mouse models, or affecting targets included among the Gene Ontology (GO) list of genes involved in angiogenesis.

### Multiplex ligation-dependent probe amplification (MLPA)

MLPA was performed to detect exonic deletions in *ENG* and *RASA1/EPHB4* using the commercial kits SALSA P093 (*ENG*) and P409 (*RASA1/EPHB4*) (MRC-Holland, Amsterdam, Netherlands). We used 100 ng of denatured genomic DNA from the patients and controls in overnight annealing of the exon-specific probes and subsequent ligation reactions. PCR was carried out with FAM-labeled primers using 10 μL of the ligation reaction. Separation was performed using an ABI Prism 3100 Genetic Analyzer (Applied Biosystems, Foster City, CA, United States), and MLPA data analyses were performed using the Coffalyser Net™ v.1 software (MRC-Holland). A reduction of the peak area value to <0.5 or increase of >1.5 was considered to indicate a deletion or duplication, respectively.

## Results

In our cohort, genetic analyses were performed for 29 patients with vein of Galen abnormalities; pathogenic/likely pathogenic variants in the VGAM/VGD genes were noted in five patients (17%), providing a clear genetic diagnosis. A VUS was found in one patient (3%) in the *EPHB4* gene, whereas variants in novel genes possibly associated with cerebrovascular disorders were reported in five out of the 29 individuals (17%). As a retrospective study, different genetic techniques were used for analyses over the 13 years of the study period (2011–2024). Four patients received a genetic diagnosis through NGS panels, and one patient was diagnosed by WES. The genetic and clinical data are summarized in Supplementary Table S1. WES analysis will be proposed as the next control to patients who have undergone only gene panel analysis.

The five patients with diagnostic genetic analyses included three men and two women. Variants in the *RASA1* gene were identified in two patients, while *EPHB4* variants were detected in two patients; one patient showed a variant in the *ACVRL1* gene*.* A description of the genotypic and phenotypic features of patients with variants in already reported VGAM/VGD genes is provided below, and a description of the five putative candidate genes is provided in the description section.

### Variants identified in *RASA1*

**FIGURE 1:**
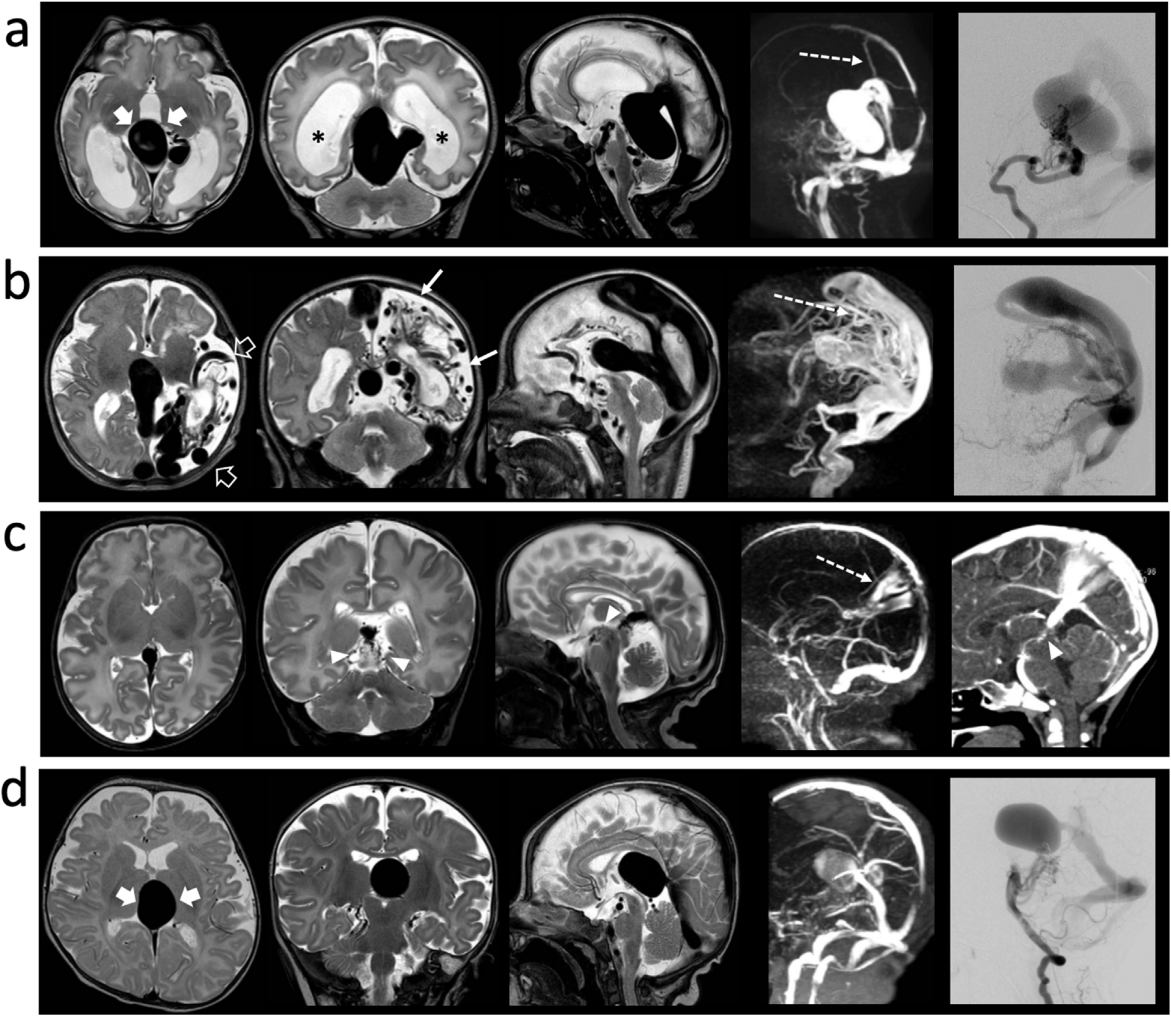
Neuroimaging features of **(A)** patient 1 and **(B)** patient 2 at birth, **(C)** patient 3 at 1.5 months of term-equivalent age, and **(D)** patient 4 at 3 months of age. Brain magnetic resonance imaging (MRI) examinations with axial T2-weighted (first column from left), coronal T2-weighted (second column from left), sagittal T2-weighted (third column from left), and venous MR angiography (fourth column from left); lateral views of **(A, B, D)** catheter angiograms (right column) and **(C)** CT angiography. Patients 1 and 4 show true vein of Galen malformations (VGAMs; thick arrows), while patient 2 shows a vein of Galen dilatation (VGD) secondary to a very large arteriovenous fistula in the right fronto-parietal region (empty arrows) and patient 3 shows a small arteriovenous malformation in the mesencephalon (arrowheads). Falcine sinuses are noted in patients 1, 2, and 3 (dashed arrows). We also note a marked ventricular dilatation with reduced white matter volume in patient 1 (asterisks) and a chronic ischemic infarct of the left hemisphere in patient 2 (thin arrows) that were present at birth.

Patient 1 is a 11-year-old boy harboring the pathogenic variant c.656C>G (p.Ser219*) that he shared with his mother; he only showed cutaneous CMs but had normal brain MRI scans. The maternal family history of this patient is significant, and the mother’s father and three uncles were described to have cutaneous angiomas, where two of them died of stroke before the age of 50 years. The following pregnancy in the mother was interrupted due to diagnosis of cerebral AVM and encephalomalacia in the fetus, who had inherited the maternal variant. In patient 1, a cerebral vascular abnormality was identified by fetal ultrasound during the third trimester, which was confirmed through fetal MRI and brain MRI at birth, and both methods confirmed the presence of a VGAM (Figure 1A). Marked supratentorial ventricular dilatation was also found, and the patient underwent embolization at 9 days of life that resulted in severe post-treatment hemorrhage with hydrocephalus requiring a ventriculoperitoneal shunt. The neurological outcome was severe, with spastic quadriplegic cerebral palsy, minimally conscious state, and refractory epilepsy (POPC 4). The child currently presents 14 cutaneous CMs that are spread over his trunk and limbs.

Patient 2 is a 6-year-old girl. Pregnancy was achieved with sperm donation for azoospermia. Prenatal ultrasound during the third trimester showed dilatation of the vein of Galen and cardiomegaly. The diagnosis of VGD was then confirmed by fetal MRI and brain MRI at birth, which showed an AVF draining into a dilated vein of Galen and superior sagittal sinus. Bilateral subacute white matter ischemic changes and chronic left fronto-temporo-parietal ischemic infarcts were also observed (Figure 1B). Three embolization procedures were necessary in the newborn period for high-output heart failure. The girl presented delayed psychomotor milestones; head control was achieved at 12 months of age, and walking with support and first words were achieved at 25 months. The patient currently shows right hemiplegia, walks with unilateral support (POPC 3), and is on enteral nutrition via gastrostomy. She currently presents seven cutaneous CMs on her trunk, left arm, and lower limbs. The variant has not been identified in the mother, and NGS analysis highlighted the *de novo* frameshift variant c.1986_1989delAAAG (p.Lys664Alafs*13) in the *RASA1* gene*.* This variant was previously unreported, has an extremely low frequency in gnomAD, and is predicted to have a functional effect on the non-sense mediated decay processes of messenger RNA, which have already been reported in literature as disease-related mechanisms (Revencu et al., 2013b) and considered pathogenic according to ACMG guidelines.

### Variants identified in *EPHB4*

Patient 3 is a 3-year-old girl born to healthy non-consanguineous parents with unremarkable family history. At 31 weeks of gestation, severe hydrops was noted along with pleural and pericardial effusions, which necessitated urgent birth through caesarian section. The newborn girl had APGAR scores of 3 and 4 and was admitted to the intensive care unit. A karyotype analysis showed negative result, and a small VGAM was suspected from brain ultrasound. Brain MRI at term-equivalent age demonstrated a small AVM originating from the posterior cerebral arteries and draining into an enlarged vein of Galen (VGD) (Figure 1C).

Psychomotor development was within the normal range, and the child is currently in good health (POPC 1). NGS analysis highlighted the c.2231G>A (p.Arg744His) variant in *EPHB4*, which has been classified as likely pathogenic based on previous associations in two cases of fetal hydrops and experimental functional evaluation for causality (source: NCBI). This missense variant is absent in the gnomAD exomes/genomes databases, and the corresponding amino acid residue (Arg744) is included in the active site of the mature protein (source: UniProt). Unexpectedly, a segregation study confirmed that the variant was inherited from her healthy father, who has no apparent clinical manifestations, suggesting incomplete penetrance and/or other contributing genetic factors.

Moreover, the variant (c.3G>A; p.Met1?) in the *EPHB4* gene was found in patient 10 through singleton WES analysis. In this case, the inheritance is unknown. The variant was absent in ClinVar and in available databases; it affects a conserved amino acid of the protein and was considered LP according to the ACMG guidelines. Clinical familial anamnesis was unremarkable; the child presented with genuine VGAM that was prenatally identified and underwent embolization at birth. The patient is now 17 years old, in good health, and is reported to have only mild deficit attention and dysgraphia.

### Variant identified in *ACVRL1*

Patient 4 is a 11-year-old boy born to second-degree cousins who are both in good health. The family history was positive for a second-degree cousin with mild developmental delay. Fetal ultrasound during the last trimester revealed a VGAM, so a Cesarean section was planned at term. The newborn showed APGAR scores of 7 and 8, with mild hypotonia. The diagnosis of VGAM was confirmed by brain MRI (Figure 1D). At 3 months of age, the infant underwent embolization, and no problems were reported regarding the psychomotor development, learning abilities, or behavior. During clinical genetics evaluations, the subject was noted to have only very mild motor clumsiness with scoliosis, flat feet, and sandal gap. The child is currently in good health, without any signs of vascular issues or bleeding. No episodes of epistaxis were reported. Brain MRI at 9 years of age showed a cavernoma in the left middle cerebellar peduncle, which had increased in size at follow-up scans performed at 10 and 11 years of age.

NGS highlighted the presence of the missense variant c.773G>A (p.Gly258Asp) in the *ACVRL1* gene. Segregation analysis was negative, and the variant was previously not described; computational prediction tools unanimously support a deleterious effect on the gene. In addition, this is a missense variant in a gene with a low rate of benign missense mutations, for which missense mutation is a common mechanism of a disease (source: Franklin); hence, the variant is considered to be LP.

### VUS identified in VGAM/VGD genes

A VUS in *EPHB4* was identified in patient 11 through singleton WES analysis (c.874A>G; p.Ser292Gly). The patient presented with true VGAM, for which he underwent embolization at 6 months of age; the child is now in good health. The patient’s family history shows a paternal uncle who underwent surgery for non-well-specified aortic valve pathology. This variant is extremely rare in the general population and has not been reported in ClinVar. However, it affects a well-known VGAM/VGD gene, based on which we postulate a significant role in the clinical phenotype of patient 11.

## Discussion

### Variants in VGAM/VGD-related genes and genotype–phenotype correlations

**FIGURE 2:**
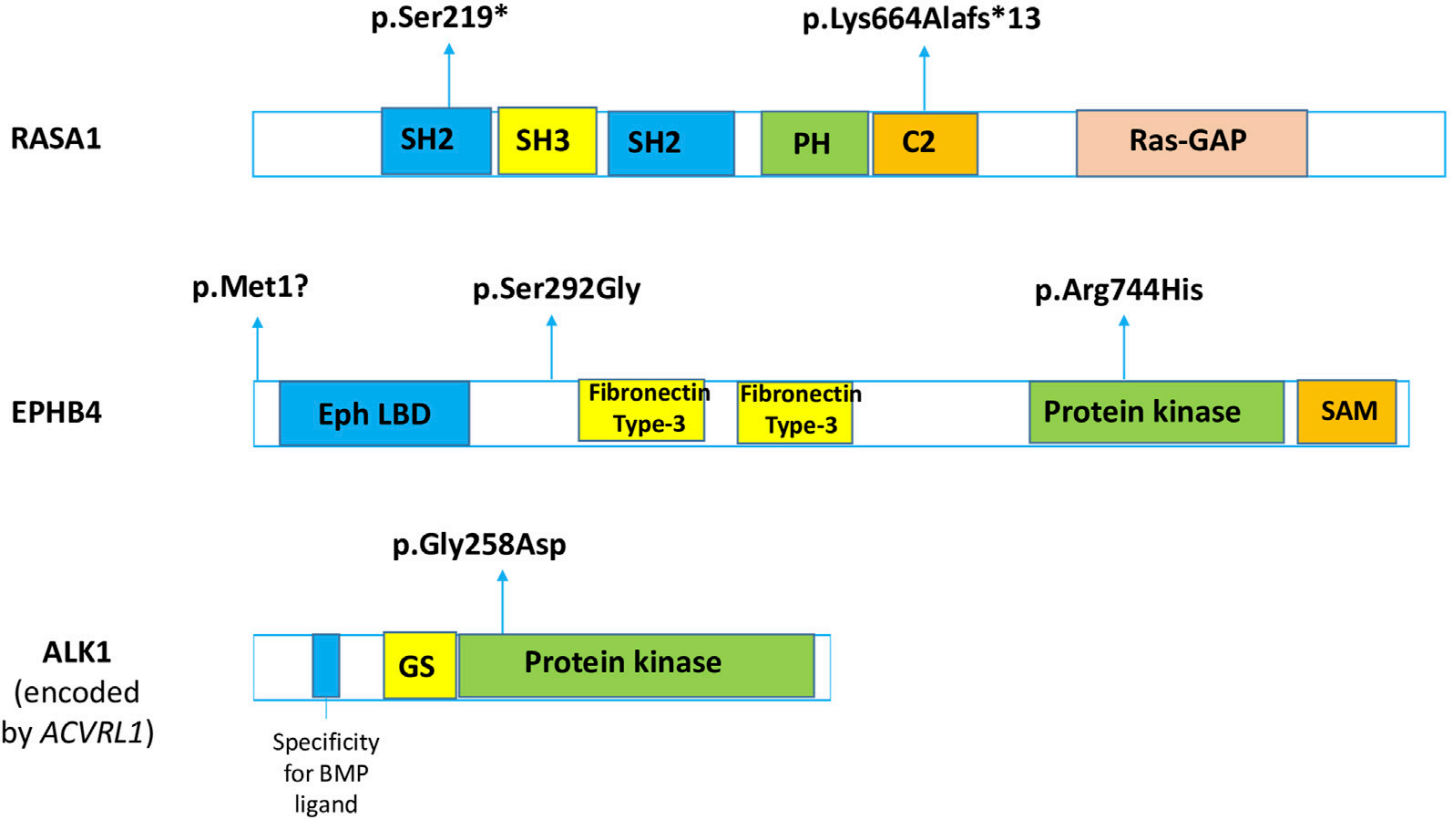
Identified variants in the VGAM/VGD genes mapped along the respective protein sequences. The functional domains are also shown. SH, Src homology; PH, pleckstrin homology; C2, Ca(2+)-binding motif; Ras-GAP, Ras-specific GTPase-activating domain; LBD, ligand-binding domain; SAM, sterile alpha motif; GS, glycine- and serine-rich sequence; VUS, variant of unknown significance. Adapted from UniProt and Zhao et al. (2023).

In our cohort, five patients with either true VGAM (n = 3) or VGD (n = 2) were identified with disease-related variants, thus receiving definite genetic diagnoses; further, two patients presented *RASA1* variants, three showed *EPHB4* variants, and one patient showed an *ACVRL1* variant (Figure 2). Some genotype–phenotype correlations were confirmed through our analyses: patients 1 and 2 who harbored *RASA1* mutations respectively presented with true VGAM and AVF, in line with recent evidence about *RASA1* carriers (Tas et al., 2022). However, the others diverged from current literature, suggesting specific roles of *EPHB4* in true VGAM, *ACVRL1* in pial AVFs (Tas et al., 2022), and *RASA1* variants more frequently described in VGAM patients (Bayrak-Toydemir et al., 2011). Patient 3, who was identified to have an *EPHB4* variant, was affected by VGD due to AVM; patient 4, who had a likely pathogenic variant in the *ACVRL1* gene, presented with true VGAM. These findings are supported by careful neuroradiological revisions and classifications and highlight the difficulties in inferring clear genotype–phenotype correlations in patients with vein of Galen abnormalities. The identification of possible distinctive genetic backgrounds between VGAM and VGD may be crucial for a neuroradiological differential diagnosis, which would also support different treatment strategies. Differential diagnoses of these rare entities are challenging, especially when physicians do not deal with them routinely or cases are reported at centers that are not highly experienced in the field.

No clear genotype–phenotype correlations in VGAM/VGD have been inferred in most of the recent case series reported in literature (Zhao et al., 2023). According to current literature, variants in *EPHB4* seem to be specifically related to VGAM, while germline *RASA1* variants are mostly found in both pial/choroidal fistulas and in VGAM patients. However, *ACVRL1* was considered to be specific to pial AVFs (Tas et al., 2022). Moreover, the terms describing “VGAM” and “pial or choroidal shunts draining into the vein of Galen (VGD)” are often used mutually in literature, without specifying the characteristics of the veins draining the shunt (Tas et al., 2022). In reality, true VGAM should be distinguished from pial and/or choroidal arteriovenous shunts draining into the vein of Galen by the type of venous drainage. In the case of VGAM, the drainage is anomalous, and the vein of Galen (specifically the embryonic venous precursor) drains only the shunt; deep drainage of the normal brain is achieved through alternative venous routes, including the latero-mesencephalic and latero-pontic veins toward the higher petrous sinuses. Instead, for choroidal or pial AVFs flowing into the vein of Galen (VGD), there is competition between abnormal and normal venous drainage. Hence, endovascular occlusion of the vein of Galen in VGAM is believed to allow occlusion of the malformation. However, Tas et al. (2022) recently suggested that such occlusions may expose the patient to severe hemorrhagic or ischemic venous complications from arteriovenous shunts that are in competition with the deep cerebral venous system in the case of the VGD (not a true VGAM). More analyses and case series are thus needed to infer the putative selective roles of specific genes in the diverse forms of vein of Galen abnormalities. A detailed neuroradiological description distinguishing true VGAM from secondary VGD is mandatory for not only better pathophysiological insights but also better treatment strategies.

With respect to segregation, patients 2 and 4 harbored a *de novo* variant. The genetic diagnosis for patient 10 was based on singleton WES, so the segregation states in the parents are unknown. In two other cases, the variant was inherited from a parent; patient 3 carried the same variant (c.2231G>A) in the *EPHB4* gene as her apparently unaffected father, while patient 1 shared an *RASA1* variant (c.158C>G) with his mother that only caused minor cutaneous CMs. The inherited VGAM variants display incomplete penetrance and variable expressivity, with the variant carriers often exhibiting only cutaneous vascular lesions (Zhao et al., 2023). The reason for such high intrafamilial phenotypic variability is unknown. Vivanti et al. (2018) described the case of a child suffering from VGAM and cutaneous CMs, where the carrier mother only showed a minor cutaneous CM and absence of any neurovascular involvement. These data support the effects of other genes in the phenotypic expression (Vivanti et al., 2018) or environmental/genetic modifiers (Zhao et al., 2023). There is very scant literature available on possible modifying factors; as an example, we cannot exclude that the risks of differential hemorrhage may be partially attributable to possible coagulation defects/mutations of the genes encoding coagulation factors. A two-hit mechanism model of pathogenesis has been described earlier, where the phenotypic expression relies on an inherited germline mutation along with a secondary postzygotic (somatic) mutation in a second allele (Zhao et al., 2023); in a recent work, a somatic inactivating *RASA1* mutation was additionally identified in a CM lesion tissue in a patient harboring a germline *RASA1* mutation, which provides evidence for the above hypothesis (Vivanti et al., 2018; Lapinski et al., 2018).

Other useful aspects emerge from the case analysis of patient 4, who harbors a likely pathogenic *ACVRL1* variant. Mutations in *ENG* and *ACVRL1* have been identified in conditions where VGAM has possible associations. These genes play critical roles in the TGF-β signaling pathways during proper vascular development, and the mutations contribute to the pathogenesis of VGAMs (Duran et al., 2018; Singh et al., 2022). Genetic analyses of *ACVRL1*, *ENG*, and *SMAD4* (in addition to *EPHB4* and *RASA1*) are considered reasonable in all cases of high-flow intracerebral vascular malformations regardless of other HHT signs (Tas et al., 2022; Revencu et al., 2013b). Monoallelic mutations of the *ACVRL1* gene encoding a cell-surface receptor for the TGF-β superfamily of ligands and expressed on endothelial cells are known to be responsible for HHT (Vivanti et al., 2018; Duran et al., 2018); they are also reported to be associated with isolated VGAM. One example of this is the case of a child who is only affected by VGAM and shares the c.652C>T variant in *ACVRL1* with his unaffected mother and elder sister, suggesting autosomal dominant heritability with low penetrance (Chida et al., 2013). De Luca et al. (2020) reported the first case of prenatal HHT diagnosis based on the presence of VGAM in a fetus that harbored the c.1451G>A variant in *ACVRL1*; the same variant was also present in other members of the family, who showed different signs of the HHT spectrum (not VGAM), suggesting significant phenotypical heterogeneity even within the same family (De Luca et al., 2020). Patient 4 was affected by isolated VGAM and cerebral cavernoma, which further supports the role of *ACVRL1* in VGAM development regardless of other signs/symptoms of HHT (Chida et al., 2013); however, it is important to remember that other HHT signs (mucocutaneous telangiectasias and epistaxis) may appear later. Intriguingly, we noted the presence of a cerebral cavernous angioma in patient 4: in a recent study analyzing the complex distribution of cerebrovascular phenotypes in HHT patients, two individuals separately carrying the c.1231C>T and c.771_772dup variants in *ACVRL1* were described to have cavernomas. However, other patients of the cohort harboring the c.1231C>T variant were not affected, suggesting high phenotypic heterogeneity among carriers of the same mutation (Gaetani et al., 2022). Even though the reason for the presence of cavernous angiomas in a minority of the *ACVRL1* mutated patients remains unknown, our evidence supports a possible connection. Further studies are therefore needed to substantiate this finding.

### Variants affecting vascular physiology identified by WES

We provide preliminary data on five genes that have not been associated with VGAM/VGD previously, which display important functions in vascular development and homeostasis. These genes require further studies to confirm their possible involvements in vascular congenital anomalies. Only functional studies supporting effects on vascular malformations will make them addressable for genetic diagnosis.

In patient 5, the diagnosis was delayed, and brain MRI as well as MRA performed at 2.5 years of age revealed a VGAM associated with an extended fine network of thalamic collaterals and supratentorial hydrocephalus. The patient underwent embolization, with total recovery of neurological symptoms and signs. WES analysis highlighted the *de novo* monoallelic variant c.1069del that introduced a premature stop codon (p.Ala357Profs*52) in the *ANTXR2* gene; this variant was reported in ClinVar in patients with hyaline fibromatosis syndrome. In fact, biallelic *ANTXR2* variants have been known to cause hyaline fibromatosis syndrome (OMIM #228600). This gene encodes a receptor for anthrax toxin, and its normal physiological functions are only partially understood. The protein is involved in extracellular matrix adhesion, where it binds to collagen IV and laminin. Anthrax toxin receptor 2 (ANTRX2) is also known as capillary morphogenesis gene 2 (CMG2) and has emerged as a crucial factor in the proliferation and morphogenesis of endothelial cells (Reeves et al., 2010). ANTXR2/CMG2 is expressed in the endothelial cells of different tissues in mice, including skin, colon, and lungs, as well as in human breast tissue. The expressions of ANTXR2/CMG2 in the endothelial cells of mouse and human tissues are consistent with its functions in angiogenesis. RNA interference experiments were performed to achieve significant reduction of ANTXR2/CMG2 expression in cultured human umbilical venous endothelial cells; this reduced expression resulted in significant inhibition of proliferation and reduced capacity to form capillary-like networks *in vitro*. Overexpression of ANTXR2/CMG2 was demonstrated to have the opposite consequences (Reeves et al., 2010). More recently, ANTXR2/CMG2 has been implicated in the migration of endothelial cells toward a growth factor gradient. Knockout/inhibition of the physiological ligands binding to CMG2 significantly decreases neovascularization and cell migration in response to growth factors (Cryan et al., 2022). Pathogenic variants of *ANTXR2* have never been reported in association with congenital vascular malformations. Nevertheless, given the crucial role played by this gene in angiogenesis, we cannot exclude a possible monoallelic involvement of the c.1069del frameshift variant in the vascular phenotype presented by the patient.

Another finding of possible clinical interest in our cohort was the c.110A>C (p.Lys367Thr) variant in *HABP2* observed in patient 6. Brain MRI and MRA performed at birth showed a choroidal-type VGAM. According to the ACMG guidelines, this variant is classified as a VUS and has an extremely low frequency in the gnomAD population database. *HABP2* is associated with non-medullary thyroid cancer (OMIM #616535) and susceptibility to venous thromboembolism (OMIM #188050) in an autosomal dominant fashion. *HABP2* or hyaluronic acid binding protein 2 is also called factor VII activating protease (FSAP) and encodes a serine-protease activating coagulation factor VII. Moreover, HABP2 is involved in the inhibition of proliferation and migration of vascular smooth muscle cells (Kannemeier et al., 2004) as well as inhibition of neointima formation in a mouse vascular injury model (Sedding et al., 2006). Hence, we speculate a potential role of this variant in the pathogenesis of the vascular malformation observed in the patient. However, the possible association with the vascular phenotype requires further analyses and validations.

In patient 7, brain MRI and MRA were performed at birth and depicted a VGAM associated with the pseudofeeders of the left middle cerebral artery. The c.1183C>A (p.Pro395Thr) variant of the *ELN* gene was identified as the cause, which was shared with the apparently healthy father (no paternal brain MRI is available); this is considered as a VUS according to ACMG guidelines. The variant has a very low frequency in gnomAD and is predicted to be deleterious by the bioinformatics tool AlphaMissense. Monoallelic variants of the *ELN* gene are known to be responsible for autosomal dominant cutis laxa (OMIM #123700) and supravalvular aortic stenosis (OMIM #185500). None of these issues were described in the patient. However, the *ELN* gene encodes for elastin, which is a key regulator of vascular remodeling. The elastic fibers are responsible for vessel elasticity and resilience that are necessary for absorbing hemodynamic stress (Wong et al., 2015). Polymorphisms in the *ELN* gene are known to contribute to the pathogenesis of aortic aneurysms and arterial dilatations (Saracini et al., 2012). Therefore, we speculate that the newly identified variant in the *ELN* gene may be a part of the vascular phenotype observed in the patient.

Patient 8, who was affected by true VGAM, harbors the c.1422_1430del (p.Ala476_Gly478del) variant in the *EPOR* gene, which is considered a VUS according to ACMG guidelines. This variant is absent in gnomAD and is characterized by protein length changes that cause in-frame deletions/insertions in a non-repeat region or a stop-loss variant. However, this variant was observed in the homozygous state in population databases more often than for disease states. *EPOR* variants have never been described in association with VGAM; *EPOR* encodes the erythropoietin (EPO) receptor, which is a member of the cytokine receptor family. Upon binding EPO, this receptor activates different intracellular pathways via Jak2 tyrosine kinase. In addition to its key role in erythropoiesis, EPO promotes neovascularization by facilitating proliferation and migration of endothelial cells as well as matrix metalloproteinase 2 (MMP2) production. In a recent work, EPO/EPOR signaling was considered to be evidence as an essential mediator of angiotensin-II-induced abdominal aortic aneurysm in *Apoe* ^−/−^ mice (Zhang et al., 2021). Thus, we cannot exclude a contributory role of *EPOR* to the congenital vascular malformation exhibited by the patient, and this possible association needs further investigations.

The c.13428G>A (p.Ala4476=) variant of *RNF213* was identified in patient 9, who had true VGAM. Successful pregnancy was achieved after intracytoplasmic sperm injection (ICSI) with both oocyte and sperm donation. Although this variant results in a synonymous amino acid change in the C-terminal of the protein, it is considered a VUS as per ACMG guidelines using some prediction tools (SpliceAI score: 0.49 moderate, dbscSNV Ada score 1 deleterious) that support a deleterious effect, probably because it could generate alterations in the splicing of the exon 52. Variants in the *RNF213* gene are responsible for a spectrum of cerebrovascular disorders, like moyamoya disease type 2 (OMIM #607151), intracranial aneurysms, and AVMs. Although the roles of the *RNF213* gene in vascular anomalies are poorly understood, the associations of different variants with different kinds of vascular disorders have been established, especially in Asian patients. Functional studies were performed to gain better insights into the possible angiogenic roles of *RNF213*; in hypoxia, mice harboring the *Rnf213* (p.Arg4757Lys) variant corresponding to human *RNF213* (p.Arg4810Lys) showed inhibition of angiogenesis instead of the pro-angiogenic effects noted in wild-type mice (Kobayashi et al., 2015). This study demonstrates the role of *RNF213* in angiogenesis (Liu et al., 2021). Although there are no reported cases of VGAM/VGD patients with *RNF213* variants, the role of the c.13428G>A variant identified in patient 9 can be postulated. More studies and VGAM/VGD case series are therefore needed to infer better correlations.

GO enrichment analysis was performed on the list of identified genes (*RASA1, EPHB4, ACVRL1, ANTXR2, HABP2, ELN, EPOR,* and *RNF213*) to confirm possible enrichments in the biological processes linked with vascular homeostasis. Statistically significant enrichments were reported in the biological processes “sprouting angiogenesis” and “circulatory system development” (source: Gene Ontology) in line with the hypothesis of our study. However, no interactions were detected in the STRING database, except for mutual interactions between *EPHB4* and *RASA1* (combined confidence of the functional interaction stated as very high) and between *RASA1* and *ALK1* (encoded by *ACVRL1*; combined confidence of the functional interaction considered to be of medium grade in STRING).

### Limitations of the study

The main limitations of this study are the retrospective design and small sample size. Different patients were evaluated using different techniques over the years, reflecting the crucial role played by the current introduction of WES analyses in clinical practice. We note that in 18 out of the 29 patients, no useful genetic findings were evident. This may be attributed to different reasons. First, the genetics of VGAM/VGD is not fully elucidated; some yet-unknown genetic or epigenetic mechanisms could contribute to the phenotypes described herein. Second, as previously stated, not all patients underwent WES analysis, which could be used to elucidate possible additive variants. Third, it is possible that variations may occur in the intronic or other regions involved in gene expression that are not evaluated through NGS. Statistical analyses on such a small sample pool are inadequate at best. VGAM is a rare disorder, and multicenter studies are essential to obtain meaningful results, especially when complex genetic studies are involved. The number of genetically diagnosed patients in our study were few since genetic analyses were unavailable, pending, or programmed (patients 12–29) for the majority of patients in our cohort (29 individuals).

### Future perspectives

Promising approaches for future investigations include RNA-sequencing studies and somatic WES analysis to yield post-zygotic variants confined to the affected tissues. Novel studies along with systematic comparisons of phenotypic data from VGAM/VGD case series will also support gene discovery through clustering of cases with similar phenotypes, thereby allowing definitions of clinically homogeneous subclasses. This may have positive effects in terms of better clinical management, treatment options, and family counseling (Chida et al., 2013). Genetic evaluations should be particularly careful and include detailed evaluations of the parents as well as complete pedigree collection; this is because variants can be inherited from a mildly affected parent in many cases, which will have important consequences for familial management and follow-up.

## Conclusion

VGAM/VGD can manifest in the context of Mendelian disorders affecting vascular development; these are characterized by variable expressions and incomplete penetrance. In this study, we analyzed genotypic and phenotypic data from a retrospective cohort of 29 VGAM/VGD patients who were treated at a tertiary care center. WES analysis will be performed on patients who are yet to receive a genetic diagnosis. In seven of the cases, variants were identified in already reported VGAM/VGD genes, including *EPHB4, RASA1*, and *ACVRL1*; thus, the genetic diagnostic rate was about 17% (5 patients out of 29) and slightly higher than a recent case series; however, this was focused only on the *EPHB4* gene (Bayrak-Toydemir et al., 2011). Some differences were also evidenced in genotype–phenotype associations based on comparisons with current literature: a patient harboring a *EPHB4* variant was affected by VGD (not VGAM), and a carrier for an *ACVRL1* variant (seemingly specific for VGD with AVFs) presented with VGAM. Since our cohort of patients was recruited from a referral center for VGAM/VGD that handles a large part of the affected children in Italy, we believe that our study can be generalized to other international cohorts; we also believe that our findings are useful for both physicians and researchers handling these rare and complex entities. We present preliminary data on variants detected in five patients, where the genes are already known to have possible effects on vascular development/remodeling, for which we speculate possible connections with VGAM/VGD. Further studies along with novel case series will probably be able to better define the genetic background and different pathogenic mechanisms underlying these diverse and peculiar vascular phenotypes.

## Data Availability

The original contributions presented in the study are publicly available. This data can be found here: https://databases.lovd.nl/shared/individuals?search_created_by=04817.
